# Conceptualising spaced learning in health professions education: A scoping review

**DOI:** 10.1111/medu.14025

**Published:** 2019-12-20

**Authors:** Marjolein Versteeg, Renée A. Hendriks, Aliki Thomas, Belinda W. C. Ommering, Paul Steendijk

**Affiliations:** ^1^ Department of Cardiology Leiden University Medical Center Leiden the Netherlands; ^2^ Center for Innovation In Medical Education Leiden University Medical Center Leiden the Netherlands; ^3^ School of Physical and Occupational Therapy Montreal Québec Canada; ^4^ Faculty of Medicine Institute for Health Sciences Education McGill University Montreal Québec Canada; ^5^ Centre for Interdisciplinary Research in Rehabilitation Montreal Québec Canada

## Abstract

**Objectives:**

To investigate the definitions and applications of ‘spaced learning’ and to propose future directions for advancing its study and practice in health professions education.

**Method:**

The authors searched five online databases for articles published on spaced learning in health professions education prior to February 2018. Two researchers independently screened articles for eligibility with set inclusion criteria. They extracted and analysed key data using both quantitative and qualitative methods.

**Results:**

Of the 2972 records retrieved, 120 articles were included in the review. More than 90% of these articles were published in the last 10 years. The definition of spaced learning varied widely and was often not theoretically grounded. Spaced learning was applied in distinct contexts, including online learning, simulation training and classroom settings. There was a large variety of spacing formats, ranging from dispersion of information or practice on a single day, to intervals lasting several months. Generally, spaced learning was implemented in practice or testing phases and rarely during teaching.

**Conclusions:**

Spaced learning is infrequently and poorly defined in the health professions education literature. We propose a comprehensive definition of spaced learning and emphasise that detailed descriptions of spacing formats are needed in future research to facilitate the operationalisation of spaced learning research and practice in health professions education.

## Abstract 2

Digging into the educationally strategic phenomenon of spaced learning, the authors discover variety that leads them to propose a comprehensive definition and emphasise that detailed format descriptions are needed to facilitate further development.

## 1 INTRODUCTION

The spacing effect is one of the most robust phenomena in the science of learning. Hundreds of published reports have replicated the spacing effect, originally uncovered by Ebbinghaus, which suggests that knowledge retention is enhanced when learning sessions are spaced.^1^, ^2^ Re‐exposing learners to information over time using temporal intervals (ie spaced learning) results in more effective storage of information than if it was all provided at a single time (ie massed learning). There is mounting evidence that students do not remember what is learned, also in health professions education (HPE).^3^, ^4^, ^5^, ^6^, ^7^ Researchers have therefore indicated a need to invest time and resources in helping learners retain the information being learned.^7^ Educational principles grounded in a spaced learning approach have the potential to address this growing challenge in HPE.

Although literature reviews on effective learning in HPE exist and suggest a key role for spaced learning in optimising retention, systematic analysis of spaced learning research is complicated by the great diversity in the terms and definitions used in this literature, including ‘distributed practice’, ‘spaced education’, and ‘retrieval practice.’^8^, ^9^, ^10^, ^11^, ^12^ The variety of learning and assessment methods that are referred to as spaced learning further complicate the analysis of its effects. According to definitions used by psychologists, spaced learning should include learning sessions that are spaced over time and include repeated information.^13^ Both cumulative testing and simulation training as performed in HPE, for instance, can be considered applications of spaced learning. In addition to the variety of educational activities, spacing formats often differ in terms of their temporality, with some researchers distributing learning sessions over a few days, whereas others use hours, weeks or months. Moreover, it is often unclear if researchers used evidence from empirical research or relied on a theoretical framework to inform their spacing format. Overall, the broad range of terms associated with spaced learning, the multiple definitions and variety of applications used in HPE can hinder the operationalisation of spaced learning.

A comprehensive synthesis of the various definitions and applications of spaced learning in HPE may help identify gaps in knowledge, highlight areas for future research and support a more effective implementation of spaced learning in the HPE curricula. Therefore, the purpose of this paper was to investigate how spaced learning is defined and applied across HPE contexts.

## 2 METHODS

We employed a scoping review methodology to examine the definitions and applications of spaced learning in HPE. To execute the review in a rigorous manner, we assembled a research team consisting of co‐investigators with in‐depth knowledge of HPE (MV, RAH, AT, BWCO and PS), methodological experience (AT and BWCO),^14^ and medical library expertise (CP).

We used the methodological framework developed by Arksey and O'Malley,^14^ which was later refined by Levac and colleagues.^15^ The framework consists of the following six steps: Step 1, identifying the research question; Step 2, identifying relevant articles; Step 3, selecting articles; Step 4, charting the data; Step 5 collating, summarising and reporting the results, and Step 6, consultation. Step 6, consultation, was not conducted as we aimed to study the HPE literature specifically without including additional stakeholders’ perspectives on this matter.

### 2.1 Identifying the research question

Given our goal of identifying key concepts, and applications of spaced learning, we generated a main research question that allows for a broad exploration of spaced learning. The overarching question guiding this scoping review was as follows: ‘How is spaced learning defined and applied in HPE?’ Accordingly, we sought to answer the following specific research questions: (RQ1A) Which concepts are used to define spaced learning and associated terms? (RQ1B) To what extent do these terms show conceptual overlap? (RQ2) Which theoretical frameworks are used to frame spaced learning? (RQ3) Which spacing formats are utilised in spaced learning research?

### 2.2 Identifying relevant studies

A university affiliated librarian (CP) was consulted when drafting the search query. An initial brainstorming session with the research team and librarian led to the inclusion of ‘spaced learning’ and possible associated terms, such as ‘spaced training’, ‘spaced education’, ‘distributed practice’, ‘test‐enhanced learning’, and ‘retrieval practice’. The final search was conducted on 28 February 2018 using five databases: PubMed, Web of Science, Embase, Education Resources Information Center (ERIC), PsycINFO (Data S1). MV conducted additional forward reference searching of included review articles to identify additional articles.

### 2.3 Selecting the studies

There was no restriction on year of publication; therefore, all articles published up until 28 February 2018 were screened for eligibility. To be included, articles had to: (a) focus on HPE (eg medicine, nursing, pharmacology), and (b) explicitly name ‘spaced learning’, or any associated term with a spaced study format. We excluded editorials, commentaries, conference abstracts and books, as well as non‐English articles.

Two researchers (MV and RAH) tested the inclusion criteria on a 10% subset of titles.^16^, ^17^ A single calibration exercise was sufficient for the team to reach full agreement after inclusion criteria were discussed and clarified. In the abstract screening stage, RAH and MV tested the inclusion criteria using a subset of papers (5%). After reaching full agreement, MV independently screened the remaining abstracts. Two additional calibration exercises were performed with RH independently screening 2.5% of abstracts (n = 34) halfway and again 2.5% (n = 34) at the end of the process to ensure that MV's interpretation of the inclusion criteria was consistent with the original calibration outcome. Disagreements were resolved by discussion. If the focus of the article was unclear based on the title and abstract, the full article was inspected.

### 2.4 Charting the data

The data charting form was developed by MV and RAH based on the units of analysis included in the research questions (eg definition, theoretical framework, timing of events and setting) using Microsoft® Excel 2010 (Microsoft Corp., Redmond, WA, USA). They independently extracted data from five full text articles to pilot the form. The usability of the charting form was discussed and minor modifications were made accordingly (ie extraction categories were added and others were removed). For instance, the ‘intervention design’ category from a previous version of the charting form was merged with the ‘timing of events’ category in the final version. The process was repeated with an additional five full text articles, followed by discussion, resulting in a final extraction form comprised of the following categories: title; author; publication year; location; terms used for spaced learning; definition by researchers; theoretical framework; population; research method; research design; report of evidence‐based spacing; timing of events; topic of learning; type of knowledge; setting; basic sciences/clinical, and learning phase.

### 2.5 Collating, summarising and reporting the results

#### 2.5.1 Numerical analyses

We performed a numerical analysis to describe the study characteristics (ie year of publication, location, population, educational content, domain, subject), theoretical frameworks (RQ2A) and spacing formats (RQ3A) included in each paper.

#### 2.5.2 Thematic analyses

The variety of spaced learning definitions and associated terms (RQ1A) were synthesised using a thematic analysis. Two researchers (MV, RAH) generated a list of open codes from words or phrases in the definitions. Discussion between the two researchers explored relationships between open codes across definitions, which we refer to as concepts. These concepts were then analysed to generate overarching core themes. Drawing from the previously identified core themes as predetermined categories, we used a deductive approach to search for conceptual overlap amongst terms and definitions (RQ1B). Cross‐checking of coding strategies and interpretation of data was performed by BWCO.

## 3 RESULTS

### 3.1 Descriptive summary

**Figure 1:**
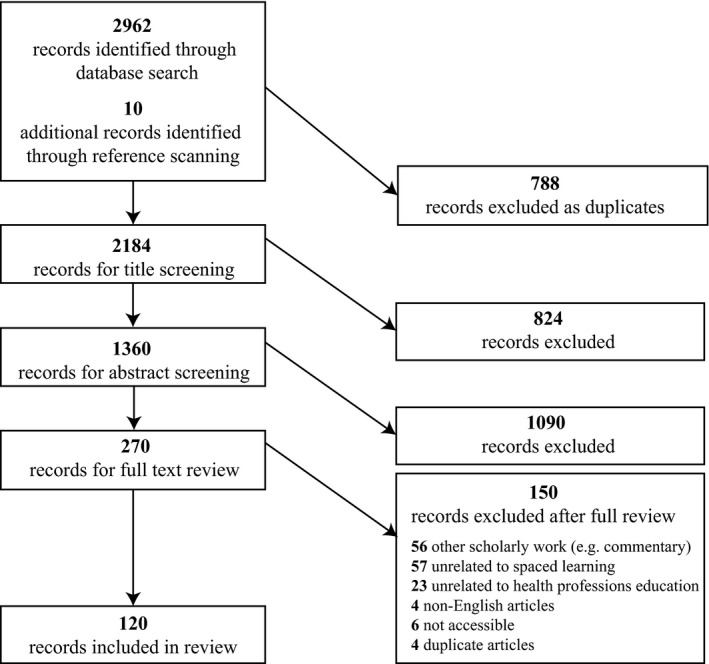
Flow chart for the scoping review selection process

The database search resulted in a total of 2972 records (Figure 1). After duplicates were removed, 2184 records remained. After applying title and abstract screening criteria, we identified 270 articles as eligible for full text review. A total of 120 articles met all criteria and were retained for the full review. Of these articles, 109 (91%) were published in the last 10 years (Data S2). Approximately two‐thirds of all studies (n = 76; 63%) were conducted in the United States, 25 in Europe (20%), eight in Canada (7%), seven in Australia (6%), two in Asia (2%) and two in South America (2%). See Data S3 for an overview of the other study characteristics.

### 3.2 Definitions of spaced learning

Besides the term ‘spaced learning’, we found 20 associated terms used to define this concept. Some terms were found in multiple studies but were defined differently (eg distributed practice), others were only defined in a single study (eg spaced distribution) or not defined at all (eg spaced retrieval practice). There was a total of 74 definitions (for an extended overview of all definitions see Data S4). These definitions were analysed thematically, resulting in the identification of seven core themes: *Educational activity* was the most recurrent theme (64/74); followed by *Structure* (51/74); *Timing* (44/74); *Content* (28/74); *Repetition* (27/44); *Learning outcomes* (24/74), and *Educational tool* (14/74). For each core theme, large variation was found amongst definitions, which resulted in a number of sub‐themes (see Table 1). For instance, an ‘educational activity’ was described in terms of what it should entail (eg listening and rereading practicing), or what it should not entail (eg not highlighting, not summarising and not cramming). Additionally, some definitions encompassed specific details about the number of educational activities and the size or the division of labour.

**Table 1:** A thematic analysis of definitions of spaced learning and its related terms

First level theme (core theme)	Second level theme	Third level theme	Fourth level theme
Educational activity	Number	Singular	
		Plural	
	Type	Reviewing	
		Reading	
		Test	Short
			Multiple choice question
			Mastery
			Physical
			Not achievement
		Distractor	
		Listening	
		Relearning	
		Case‐based	
		Receiving feedback	
		Practicing	
		Studying	
		Learning	
		Recalling	
		Not rereading	
		Not relistening	
		Not highlighting	
		Not summarising	
		Not cramming	
	Size	Curricula	
		Smaller	
	Division of labour	Providing	
		Strategy for learning (student‐directed)	
Structure	Dispersion		
	Alternation	Irregular	
		Large	
	Interruption of activity	Rest	
	Not packed together	10‐20 minutes	
	Not a single time		
	No dispersion		
	Adaptive		
Content	Information and Content	Multiple sets	
		Small	
		Identical	
		New	
	Stimuli		
Repetition	Rehearsal	Three times periodically	
Timing	Comparative	Longer	
		Later	
	Adjective	Long	
		Short	
		Fast	
		Fixed	
	Specific duration	Days	
		Weeks	
		Months	
		Less than 5 minutes	
	Other	Increasing	
		Previous	
		Immediately prior	
		Concurrently with an activity	
Educational tool	Multi‐source		
	Owned by student		
	Electronic		
	Online		
	Gamifications		
Learning outcomes	Knowledge		
	Skill		
	Impact on behaviour		
	Forgetting	Natural	
	Recall and remember	Retention	
		Silent	
	Effect	More effective	Deliberate
			Adaptive
		Reducing	

Due to this large variation in definitions, a deductive approach was necessary to study conceptual overlap between terms and this approach was conducted on the core theme level. The recurrent core themes for each of the 21 terms are shown in Table 2. For instance, for the term ‘spaced learning’ we found five definitions all of which included a notion of a certain educational activity, structure and timing.

**Table 2:** Overview of the identified core themes in the definitions

	Educational activity	Structure	Timing	Repetition	Content	Educational tool	Learning outcomes
Plural definitions							
Spaced learning (5)^71^, ^75^, ^79^, ^82^, ^127^	5/5	5/5	5/5	4/5	3/5	1/5	2/5
Spaced practice (2)^57^, ^128^	2/2	2/2	2/2	1/2	1/2		1/2
Retrieval practice (6)^12^, ^59^, ^128^, ^129^, ^130^, ^131^	6/6	1/6	3/6		1/6		2/6
Distributed practice (19)^12^, ^46^, ^60^, ^67^, ^68^, ^73^, ^88^, ^91^, ^92^, ^93^, ^99^, ^101^, ^103^, ^104^, ^106^, ^110^, ^128^, ^132^, ^133^, ^134^, ^135^	19/19	17/19	14/19	2/19	5/19		4/19
Spaced education (19)^19^, ^20^, ^22^, ^23^, ^24^, ^27^, ^28^, ^29^, ^30^, ^34^, ^35^, ^36^, ^44^, ^47^, ^52^, ^53^, ^54^, ^78^, ^81^, ^85^, ^114^, ^136^, ^137^	15/19	15/19	8/19	9/19	8/19	11/19	7/19
Single definition							
Spaced approach^72^	X	X	X				
Distributed training^98^	X	X	X				
Spaced distribution^34^, ^35^, ^36^, ^38^	X	X	X	X			
Distributed study^138^	X	X	X	X	X		X
Spaced repetition^116^	X	X	X	X	X		X
Automated spaced repetition^18^	X	X	X	X	X		X
Repeated practice^68^	X	X					
Structured spaced training^86^	X	X					
Interleaved practice^21^	X	X			X		
Spaced training^139^	X	X					X
Interactive‐spaced education^140^	X	X		X	X	X	X
Space repetition learning^45^	X		X		X		X
Interval learning^137^	X		X	X	X		
Interval training^141^	X	X	X	X			
Repeated testing^117^, ^118^	X		X	X	X		X
Distributed method of learning^105^	X		X	X	X	X	X

In case of terms with plural definitions, only core themes recurrent in all definitions are indicated. The number of plural definitions is indicated for each term in brackets. This table only shows the terms with definitions (21 total). Terms identified without definitions were: Dispersed learning;^50^ Distributed learning;^50^, ^60^ Repeated retrieval practice;^112^ Spaced instruction;^49^ Spaced training;^49^ Spaced retrieval practice;^66^ Spaced studying,^142^ and Spaced testing.^143^

### 3.3 Framing spaced learning

Almost half of the empirical research articles (n = 48, 47%) did not explicitly mention a theoretical framework. In total, nine theoretical frameworks were mentioned in the remaining studies of which the Spacing effect^18^, ^19^, ^20^, ^21^, ^22^, ^23^, ^24^, ^25^, ^26^, ^27^, ^28^, ^29^, ^30^, ^31^, ^32^, ^33^, ^34^, ^35^, ^36^, ^37^, ^38^, ^39^, ^40^, ^41^, ^42^, ^43^, ^44^, ^45^, ^46^, ^47^, ^48^, ^49^, ^50^, ^51^, ^52^, ^53^, ^54^, ^55^, ^56^, ^57^ (n = 40) and Testing effect^19^, ^20^, ^21^, ^22^, ^23^, ^24^, ^25^, ^26^, ^27^, ^29^, ^30^, ^31^, ^32^, ^33^, ^37^, ^39^, ^41^, ^42^, ^43^, ^51^, ^52^, ^53^, ^55^, ^58^, ^59^, ^60^, ^61^, ^62^, ^63^, ^64^, ^65^, ^66^ (n = 31) were named most often. Other frameworks were Cognitive Load Theory^50^, ^57^, ^67^, ^68^ (n = 4), Desirable Difficulties Theory^59^, ^69^ (n = 2), Retrieval hypothesis^70^, ^71^ (n = 2), Total‐time hypothesis^70^, ^71^ (n = 2), Learning Theory^72^ (n = 1), Metacognitive Theory^73^ (n = 1) and Kolb's Experiential Learning Theory^61^ (n = 1).

Only a few studies^26^, ^31^, ^32^, ^33^, ^34^, ^39^, ^40^, ^41^, ^56^, ^60^, ^64^, ^69^, ^74^, ^75^, ^76^ (n = 15, 15%) based their spacing format on previous empirical research. Articles by Cepeda and colleagues^13^, ^26^, ^31^, ^32^, ^33^, ^39^, ^41^, ^74^ (n = 7) and Pashler and colleagues^26^, ^31^, ^32^, ^33^, ^39^, ^40^, ^41^, ^77^ (n = 7), both derived from psychological literature on the spacing effect, were cited most often.

### 3.4 Applying spaced learning

Approximately half of the empirical research articles (n = 51, 48%) applied spaced learning in an online setting, mostly through delivering learning sessions in e‐mails distributed over time using electronic modules, eg, Qstream^19^, ^20^, ^21^, ^23^, ^27^, ^28^, ^29^, ^31^, ^32^, ^33^, ^34^, ^35^, ^36^, ^37^, ^38^, ^39^, ^40^, ^41^, ^42^, ^43^, ^44^, ^45^, ^47^, ^51^, ^52^, ^53^, ^54^, ^55^, ^64^, ^74^, ^78^, ^79^, ^80^, ^81^, ^82^, ^83^, ^84^, ^85^ (n = 38, 37%). Spaced learning was also implemented in simulation settings^46^, ^48^, ^53^, ^56^, ^65^, ^67^, ^68^, ^75^, ^76^, ^86^, ^87^, ^88^, ^89^, ^90^, ^91^, ^92^, ^93^, ^94^, ^95^, ^96^, ^97^, ^98^, ^99^, ^100^, ^101^ (n = 24, 23%), generally used to disperse training sessions over time to stimulate clinical skill acquisition. In total 24 studies^49^, ^50^, ^58^, ^59^, ^60^, ^61^, ^65^, ^66^, ^70^, ^72^, ^73^, ^95^, ^102^, ^103^, ^104^, ^105^, ^106^, ^107^, ^108^, ^109^, ^110^, ^111^, ^112^, ^113^ (23%) were conducted in classrooms and applied to various educational activities, ranging from repeated practice and testing of basic science mechanisms, to clinical scenarios and skill training.

The spacing formats of experimental and observational studies were analysed and summarised for the three different settings that were identified previously, that is online, simulation and classroom settings.

For the *online* setting, the duration of events showed a great variety between studies. Information or questions were distributed through online sources daily^22^, ^27^, ^28^, ^29^, ^30^, ^33^, ^34^, ^37^, ^55^, ^85^ (n = 10), every 2 days^19^, ^23^, ^24^, ^31^, ^52^, ^53^, ^64^, ^78^ (n = 8), every 3 days^41^ (n = 1)*,* weekly^20^, ^35^, ^36^, ^39^, ^40^, ^43^, ^44^, ^71^, ^80^, ^114^, ^115^, ^116^ (n = 12), every 2 weeks^45^ (n = 1), or monthly^43^, ^79^ (n = 2). In studies explicitly stating that material was not only spaced but also repeated, repetition delays ranged from various days^22^, ^55^ (n = 2), to weeks^19^, ^23^, ^24^, ^27^, ^28^, ^29^, ^31^, ^32^, ^33^, ^34^, ^35^, ^39^, ^40^, ^41^, ^42^, ^43^, ^51^, ^52^, ^53^, ^74^, ^78^, ^80^, ^84^, ^85^ (n = 24), to months^19^, ^30^, ^32^, ^33^, ^36^, ^37^, ^39^, ^40^, ^43^, ^44^, ^54^, ^64^ (n = 12). Additionally, there were large variations in the number of repetitions and intervals between repetitions.

For the *simulation* setting, studies frequently used designs in which training sessions were distributed within a single day^56^, ^75^, ^88^, ^92^, ^95^, ^100^, ^106^ (n = 7) or within a set number of consecutive days, weeks or months^56^, ^67^, ^68^, ^76^, ^86^, ^87^, ^88^, ^93^, ^95^, ^96^, ^97^, ^98^, ^99^, ^100^, ^106^ (n = 15). Notably, there were numerous differences in the number of training sessions, total training time and duration of intervals.

For the *classroom* setting, most studies described the use of interim (eg cumulative) testing^58^, ^61^, ^62^, ^65^, ^66^, ^70^, ^103^, ^107^, ^108^, ^109^, ^110^, ^111^, ^117^, ^118^, ^119^ (n = 15) to enhance long‐term retention of to‐be‐learned information. Other applications of spaced learning in the classroom involved the distribution of teaching or learning sessions over multiple days^60^, ^72^, ^112^ (n = 3), weeks^49^, ^50^, ^59^, ^73^ (n = 3), or months^73^ (n = 1). It was often unclear if sessions included repetition of material taught during preceding sessions or if each session solely consisted of new material.

Studies were mainly concerned with improving the effectiveness of learning through spacing of practice and/or testing (n = 91, 88%). Only four studies^50^, ^81^, ^113^, ^114^ (4%) focused efforts on spaced learning as a means of teaching, for example, during conventional lectures.

## 4 DISCUSSION

We conducted a scoping review to examine how spaced learning is defined and applied in HPE. Spaced learning appeared relatively new to HPE, with 90% of the articles in our review having been published only in the last 10 years. This is an interesting finding given that the first description of the spacing effect dates back to 1885 and has been a major subject of research in the educational psychology literature since.^120^ Our findings indicate that most spaced learning applications in HPE involve online learning, which may explain the later presence of spaced learning in our field.

In light of the increasing popularity of spaced learning in HPE, it is concerning that descriptions of its applications lack the necessary detail to support implementation or replication. Our review showed that in most research spaced learning is poorly defined and almost half of the studies do not explicitly mention a theoretical framework. Even fewer studies based their spacing formats on empirical literature. It is possible that these shortcomings may be linked to the presence of ‘innovators’ and ‘early adopters’ in our field. According to Rogers’ Diffusion of Innovation Theory,^121^ these groups value the trialability attribute of innovations (ie how easily potential adopters can explore your innovation), which aligns with our findings. All spaced learning studies in HPE that we analysed were conducted in authentic educational environments instead of laboratory settings. As such, the focus may be on improving educational practices and less on advancing theory or knowledge. However, this approach makes replication and follow‐up of current studies on spaced learning challenging. Clearer definitions and detailed descriptions of applications are needed for scholars and educators to improve future research and practice on spaced learning in HPE.

### 4.1 Defining spaced learning

We examined 74 definitions of spaced learning and associated terms. Concepts found amongst these definitions were organised into seven core themes: *Educational activity; Structure; Timing; Repetition; Educational tool*, and *Learning outcomes*. Most terms were defined by unique combinations of core themes resulting in low conceptual overlap between terms. Additionally, some terms seemed to relate to a more specified version of spaced learning as they contained more core themes than others. For instance, the definition of ‘spaced repetition’ includes the notion of ‘reviewing of content multiple times over optimised time intervals’, whereas ‘spaced approach’ limits itself to stating ‘the distribution of fixed teaching hours over a longer time period.’ It is important to note that the core themes were derived from a large variety of second to fourth level themes, illustrating the vagueness of definitions. For example, the educational activity as mentioned in the definition of ‘spaced distribution’, concerns the *number* of activities, whereas a definition of ‘spaced learning’ focuses on the *type* of activities (ie tests). Although they both say something about learning engagement, they differ in what information they deem relevant.

Furthermore, different definitions of the same term typically showed few recurrent core themes suggesting low conceptual overlap. For example, we found that the five definitions of the term ‘spaced learning’ shared the following core themes: *Educational activity; Structure*, and *Timing*; whereas *Education tool* was only found in one of the definitions.

Clearly, there is no unified definition of spaced learning in the HPE literature. We suggest that a more consistent use of terminology can facilitate a more systematic appraisal of future research. Based on our findings we propose the following comprehensive definition of spaced learning, which explicitly covers all involved components:

Spaced learning involves [specified] educational encounters that are devoted to the same [specified] material, and distributed over a [specified] number of periods separated by a [specified] interstudy interval, with a [specified] learning outcome after a [specified] retention interval.

These components should be clearly specified for each study on spaced learning to facilitate comparison and crosstalk between spaced learning researchers in our community.

### 4.2 Framing spaced learning

There is room for improvement regarding framing of the spaced learning concept as almost half of the articles did not explicitly frame their research using a theoretical framework. This might be related to the diversity and vagueness amongst terms used to define spaced learning, which may have complicated researchers’ search for previous empirical research and associated theoretical frameworks. These findings are illustrative of the general underuse of theory in HPE research.^122^, ^123^ Importantly, use of theory can help educators and researchers to better understand existing problems and formulate new research questions.

### 4.3 Applying spaced learning

Spaced learning is applied broadly in HPE, spanning various health professions, subjects, and educational settings (ie online, simulation and classroom). Exploring the specific details of its applications was rather challenging due to the absence of vital information on used spacing formats such as the number and duration of intervals between educational encounters, the duration of the retention interval, and the number and duration of learning sessions. We emphasise that in future research, spacing formats should be reported in detail to ensure reproducibility and generalisability of the outcomes.^124^, ^125^

During educational encounters, spacing formats mostly included spaced learning in the testing or practice phase. The occurrence of the ‘testing effect’ as the second most used theoretical framework fits this application pattern. Notably, less research is conducted on the benefits of spaced learning in the instructional phase, that is during teaching. We consider this a gap in the literature and propose that HPE may draw from the rich scientific literature on spaced learning in education and psychology to develop spaced learning formats that can optimise the retention of knowledge. Psychological and neuroscientific research findings on the mechanisms of memory formation suggest that spaced learning also works using shorter intervals.^126^ Therefore, applying spaced learning on the timescale of minutes to hours may have implications for current massed learning in classroom settings, such as conventional lectures, which still holds a prominent position in HPE worldwide. Ultimately, implementing and optimising spaced learning formats across curricula may help to prepare health professionals with a solid foundational body of knowledge.

### 4.4 Limitations

Although we attempted to be as thorough as possible, our search was limited to the selected databases, search terms and English‐written scholarly articles, which may have excluded relevant articles inadvertently. Furthermore, as a scoping review aims to investigate the nature and extent of the research topic, we did not critically appraise the included studies.

## 5 CONCLUSIONS

This scoping review has highlighted the large variety in definitions and applications of spaced learning across HPE. Based on our findings and our review of the psychological and neuroscientific literature, we offer the following recommendations to improve research and educational practice related to spaced learning: (a) define the spaced learning concept in an explicit and comprehensive manner in order to stimulate consistent application; (b) use study designs that are described thoroughly and informed by empirical research on spaced learning, related theories, and practices, and (c) further expand the spaced learning applications beyond online learning and simulation training, for example, by applying spaced learning in the instructional phase. With these recommendations, we aim to promote an enriched understanding of spaced learning and support the development of optimal spaced learning environments in HPE curricula.

## AUTHOR CONTRIBUTION

Researchers MV and RAH performed the conception and design of the work, analysis, interpretation of data, drafting and revision of the work. Researchers AT, BWCO and PS contributed to the interpretation of data, drafting and revision of the work. All authors (MV, RAH, AT, BWCO and PS) give their final approval of the version to be published and agree to be accountable for all aspects of the work.

## CONFLICTS OF INTEREST

None.

## ETHICAL APPROVAL

Reported as not applicable.
