# Supplementary material for: Conceptualising spaced learning in health professions education: A scoping review
Source: Med Educ. 2019 Dec 20;54(3):205–16. doi: 10.1111/medu.14025 (PMC7064953; doi:10.1111/medu.14025)
Supplement: Supplementary file 1 [file MEDU-54-205-s001.docx]

**S1.** The used literature search term combinations.

| **Spaced [ti]** |  | **Learning [ti]** |  | **Spaced learning [tw]** |  | **Health professions education [tw]** |
| --- | --- | --- | --- | --- | --- | --- |
| Spaced | AND | Learning | OR | Spaced training | AND | Education, Professional [Mesh] |
| Spacing |  | Memory |  | Spacing training |  | Medical educat* |
| Retriev* |  | Training |  | Spaced learning |  | Professional educat* |
| Repetition* |  | Educat* |  | Spacing learning |  | Teach* |
| Recall |  |  |  | Spaced interval training |  | Medical student* |
| Mental recall [Mesh] |  |  |  | Spaced interval learning  Memory training |  | Biomedical student*  Pharmacy student* |
|  |  |  |  | Spaced retrieval |  | Biology student* |
|  |  |  |  | Spaced retrieving |  | Dental student* |
|  |  |  |  | Retrieval practic* |  | Nursing student* |
|  |  |  |  | Mass learning |  | Undergraduate* |
|  |  |  |  | Massed learning |  | Graduat* |
|  |  |  |  | Spacing effect |  | Intern* |
|  |  |  |  | Distributed practic* |  | University |
|  |  |  |  | Spacing efficacy |  | Universities |
|  |  |  |  | Spacing practice Sessions |  | Resident*  Health professional* |
|  |  |  |  | Spacing of learning |  |  |
|  |  |  |  | Spacing repetition |  |  |
|  |  |  |  | Spacing and repetition |  |  |
|  |  |  |  | Spaced educat* |  |  |
|  |  |  |  | Spaced test* |  |  |
|  |  |  |  | Test-enhaned learning |  |  |
|  |  |  |  | Testing effect* |  |  |

Within each column all terms are searched with the Boolean operator “OR”.

**S2**. Frequency distribution of research articles on spaced learning included in this scoping review (n = 120). Articles are sorted by year.

**S3.** Characteristics of included research articles. Except for study location, characteristics were described for original research articles only (n = 103).

| Characteristic | No. (%) |
| --- | --- |
| **Study location** |  |
| United States  Europe  Canada  Australia  Asia  South America | 76 (63)  25 (20)  8 (7)  7 (6)  2 (2)  2 (2) |
| **Study population*** |  |
| Medical students  Residents  Other healthcare majors  *pharmacy, dental, biomedical sciences, health sciences and prehealthcare*  Other healthcare professions  *fellows, physician assistants, nurses, flight paramedics, family doctors, clinicians, program faculty and program directors*  Physicians | 38 (32)  27 (23)  18 (15)  18 (15)  9 (8) |
| **Educational content** |  |
| Knowledge  *factual, conceptual, procedural*  Skills  N.A. | 74 (72)  27 (26)  4 (4) |
| **Domain** |  |
| Clinical  Basic sciences  Miscellaneous | 66 (64)  9 (9)  28 (27) |
| **Subject** |  |
| Medical specialties  Guidelines, principles, quality control  Anatomy  Physiology  Molecular biology  Dental education  Miscellaneous  *Teaching, Statistics, Nutrition, Resuscitation, Pain-assessment, Physical examination, Sex, Cognitive Behavior Therapy* | 60 (58)  11 (11)  7 (7)  5 (5)  3 (3)  2 (2)  15 (15) |

**Some studies included multiple study populations.*

**S4.** Extended overview of spaced learning terms.

| **Terms used without definitions** |
| --- |
| Spaced testing^128^ |
| Spaced instruction^49^ |
| Spaced training^49^ |
| Dispersed learning^50^ |
| Distributed learning^50, 60^ |
| Spaced retrieval practice^66^ |
| Spaced studying^127^ |
| Repeated retrieval practice^118^ |

| **Terms with single definition** | **Definitions** |
| --- | --- |
| Spaced distribution^34-36, 38^ | Educational encounters that are repeated over spaced time intervals |
| Repeated practice^68^ | Practice is distributed over several sessions rather than massed as a single block of training |
| Spaced approach^73^ | Distribution of a fixed amount of teaching hours over a longer time period |
| Spaced repetition^109^ | A learning approach that focuses on reviewing content multiple times over optimized time intervals |
| Automated spaced repetition^18^ | The observation that it is easier to remember information when it is studied multiple times over a long time span, rather than studied once or a few times in a short time span. |
| Structured spaced training^86^ | Spaced training; in which training is undertaken with breaks. |
| Interleaved practice^141^ | to structure the acquisition of multiple learning sets such that learning alternates between sets. |
| Distributed training^98^ | A practice schedule in which periods of training are interspersed with rest periods. |
| Distributed method of learning^105^ | instead of learning a French word by continuously reading it off a flashcard, one could memorize it over days or weeks from different sources, for example, by reading newspapers, perusing restaurant menus, and hearing it in conversation. |
| Space repetition learning^45^ | Providing small modules of information to learners over time to overcome the normal forgetting that occurs. |
| Repeated testing^113^ | People learn and retain information better through repeated exposure. Actively retrieving content during a test strengthens retention even more |
| Distributed study^140^ | Break the material up into smaller portions that are studied over a period of days. A distributed study strategy is more effective when it includes repetition, with topics being re-visited, so as to reinforce learning. |
| Interactive spaced education^143^ | Interactive spaced education (ISE) is an online education system which harnesses the spacing effect, the psychological finding that educational material which is repeatedly presented over spaced intervals is learned and retained more efficiently. |
| Spaced training^142^ | Spaced training involves distributing learning tasks and skill acquisition practice sessions over a specified time interval, whereas massed practice occurs over the course of 1 or 2 intense and content-heavy sessions. |
| Interval learning^139^(32) | Learning over time. Seeing the presentation of new materials over time, in bite-sized chunks, and then seeing them again at a later time, particularly as a test. |
| Interval training^144^(25) | Learners practice multiple times for shorter periods in interval training |

| **Terms with plural definitions** | **Definitions** |
| --- | --- |
| #1Spaced practice^57^ | Distributing practice over time |
| #2 Spaced practice^130^ | Creating a study schedule that spreads study activities out over time. The same amount of repeated studying of the same information spaced out over time will lead to greater retention of that information in the long run, compared with repeated studying of the same information for the same amount of time in one study session |
| #1 Spaced learning^79^ | delivery of brief morsels of information repeated over time intervals, in contrast to massed learning, a bolus of information delivered all at once without breaks |
| #2 Spaced learning^75^ | Spaced learning is based on the temporal pattern of stimuli for creating long-term memories. It consists of blocks with highly condensed content that is repeated three times and interrupted by 10–20 min breaks during which distractor activities such as physical activity are performed. |
| #3 Spaced learning^82^ | Learning encounters that are ‘‘spaced’’ and ‘‘repeated over time’’. Spaced learning differs significantly from other pedagogies because it ‘‘pushes’’ short clinical casebased scenarios that take less than 5 minutes to consider to participants’ e-mail or hand-held mobile device. |
| #4 Spaced learning^71^ | Spaced learning fosters retention effects by a careful adjustment (increasing) of time-intervals between test repetitions |
| #5 Spaced learning^129^ | The insertion of distinct temporal gaps between learning episodes. This is in contrast to massed learning, which refers to learning with little or no time between learning sessions. |
| #1 Retrieval practice^59^ | Retrieval practice involves internally recalling previously learned information as opposed to rereading or relistening to information that was previously seen or heard. |
| #2 Retrieval practice^131^ | Being tested, pimped, or otherwise asked to exhibit knowledge to which one has been previously exposed |
| #3 Retrieval practice^132^ | In retrieval practice, testing is used as a method of teaching rather than a method of assessment |
| #4 Retrieval practice^133^ | Testing. There are greater gains to be realized with repetition of retrieval practice distributed over longer intervals and interleaved with demands for recall of other concepts or skills. |
| #5 Retrieval practice^12^ | “how” one spends time while learning is relevant to the learning science strategy, in contrast to “when” (distributed learning). |
| #6 Retrieval practice^130^ | Bringing learned information to mind from long-term memory |
| #1 Distributed practice^67^ | Practice sessions spaced in time |
| #2 Distributed practice^68^ | Practice is distributed over several sessions rather than massed as a single block of training |
| #3 Distributed practice^109^ | Scheduled educational sessions of learning and training interspersed with rest periods. Massed practice refers to a continuous block of training without rest. |
| #4 Distributed practice^88^ | Refers to a schedule where periods of practice are interspersed with periods of rest |
| #5 Distributed practice^73^ | Study activities with intervals |
| #6 Distributed practice^60^ | Distributed practice refers to spacing out ones’ practice or relearning materials intermittently over time, whereas massed practice refers to learning materials in one long session that often occurs immediately prior to a learning assessment (e.g., cramming). |
| #7 Distributed practice^91, 93^ | Practice interspersed with periods of rest |
| #8 Distributed practice^115^ | Content and understanding of concepts is improved when study is spaced or distributed over a period of time, compared to cramming or massed practice |
| #9 Distributed practice^92^ | A practice regime in which periods of training are interspersed with rest periods. |
| #10 Distributed practice^103^ | Knowledge or a skill is taught in multiple sessions separated in time |
| #11 Distributed practice^104^ | Material learnt in brief training workshops decays quickly over time, whereas repetition on many occasions ensures greater retention. |
| #12 Distributed practic46^7^ | Training in multiple sessions with different intervals |
| #13 Distributed practice^99, 100^ | Several training sessions |
| #14 Distributed practice^134^ | Practice sessions being distributed in time with each session consisting of repeated practice of the exact same procedure. Distribution of practice can refer both to distribution of content into several lessons and to practice sessions being spaced by time, often days or weeks, and is also termed (time) spaced or interval training. |
| #15 Distributed practice^135^ | Learning spread over a period of time. Theoretically, teaching in small proportions dispersed over time is better with respect to knowledge and skill retention compared with massed delivery |
| #16 Distributed practice^136^ | Practice sessions are distributed, either as massed or regular teaching sessions |
| #17 Distributed practice^139^ | Involving smaller practice sessions with large interspersed rest periods. |
| #18 Distributed practice^12^ | Spacing out of multiple study or practice sessions over an extended period of time |
| #19 Distributed practice^130^ | Creating a study schedule that spreads study activities out over time. The same amount of repeated studying of the same information spaced out over time will lead to greater retention of that information in the long run, compared with repeated studying of the same information for the same amount of time in one study session. |
| #1 Spaced education^78^ | The planned repeating of educational encounters over time to enhance knowledge retention. |
| #2 Spaced education^19^ | Spaced education involves spaced repetition of question based educational content, but also includes an adaptive rescheduling algorithm combined with compelling game mechanics. |
| #3 Spaced education^20^ | Spaced education is a teaching strategy that delivers educational content electronically over several weeks or months (spaced in time) in a case-based or quiz format. |
| #4 Spaced education^22^ | Improved knowledge retention when material is repeated at spaced intervals. |
| #5 Spaced education^23^ | The spacing effect is based on the idea of repeating information over spaced intervals of time compared with complete presentation at one time. |
| #6 Spaced education^24^ | Online learning that has been demonstrated to increase knowledge retention and impact on behaviour. SE involves participants receiving short multiple-choice questions and feedback via e-mail in a repeating pattern over a number of weeks. |
| #7 Spaced education^29^ | Online educational programs that are structured to take advantage of the pedagogical benefits of the spacing effect |
| #8 Spaced education^28^ | On-line spaced education programs attempt to improve knowledge retention by harnessing the pedagogical merits of the spacing effect. |
| #9 Spaced education^27^ | SPACED education is a novel form of online education based on the 2 core psychology research findings of spacing and testing effects. |
| #10 Spaced education^30^ | Online educational programs that are structured to take advantage of the pedagogical benefits of the ‘spacing effect’. |
| #11 Spaced education^31-36^ | Educational programs that are constructed to take advantage of the pedagogical merits of the spacing effect |
| #12 Spaced education^44^ | The psychologic finding that training or educational material that is spaced and repeated over time (spaced distribution) results in greater knowledge acquisition and retention of content than when the same material is presented at a single time point (mass distribution). |
| #13 Spaced education^106^ | Educational content delivered in small quantities and repeated over time, through electronic distribution |
| #14 Spaced education^81^ | A conceptual educational framework that relies on the delivery of succinct pieces of information related to a specific content area spaced out over time. |
| #15 Spaced education^52,53^ | Involves participants’ receiving short multiple-choice questions and feedback via e-mail in a repeating pattern over a number of weeks. |
| #16 Spaced education^54^ | Online educational programs that are structured to take advantage of the pedagogical benefits of the spacing effect, in which periodically repeated, educational encounters lead to improved knowledge attainment and retention compared with a single “bolus” educational opportunity. |
| #17 Spaced education^85^ | An e‑learning platform in which “information is presented and repeated over spaced intervals and is learned and retained more effectively |
| #18 Spaced education^138^ | Educational encounters are spaced and repeated over a defined period |
| #19 Spaced education^139^ | A web-based, student-directed learning tool |
